# Calcium Independent Effect of Orai1 and STIM1 in Non-Hodgkin B Cell Lymphoma Dissemination

**DOI:** 10.3390/cancers10110402

**Published:** 2018-10-26

**Authors:** Simon Latour, Isabelle Mahouche, Floriane Cherrier, Lamia Azzi-Martin, Valérie Velasco, Pierre Soubeyran, Jean-Philippe Merlio, Sandrine Poglio, Laurence Bresson-Bepoldin

**Affiliations:** 1 Department of Life and Health Sciences, University of Bordeaux, F-33076 Bordeaux, France; simon_latour@hotmail.fr (S.L.); i.mahouche@bordeaux.unicancer.fr (I.M.); floriane.cherrier@u-bordeaux.fr (F.C.); lamia.azzi-martin@u-bordeaux.fr (L.A.-M.); P.Soubeyran@bordeaux.unicancer.fr (P.S.); jp.merlio@u-bordeaux.fr (J.-P.M.); sandrine.poglio@u-bordeaux.fr (S.P.); 2 INSERM, U1218 ACTION, F-33000 Bordeaux, France; 3 Institut Bergonié, Comprehensive Cancer Centre, F-33000 Bordeaux, France; V.Velasco@bordeaux.unicancer.fr; 4 INSERM, U1053 BaRITOn, F-33000 Bordeaux, France

**Keywords:** Orai1, STIM1, calcium, migration, lymphocyte, lymphoma

## Abstract

Ca^2+^ release-activated Ca^2+^ channels, composed of Orai1 and STIM1 (stromal interaction molecule 1) proteins, are the main Ca^2+^ entry mechanism in lymphocytes. Their role in cell migration and metastasis is demonstrated in solid cancers but it remains elusive in malignant hemopathies. Diffuse large B cell lymphoma (DLBCL) is characterized by the dissemination of neoplastic B cells throughout the organism which is under the control of chemokines such as Stromal Derived Factor 1 (SDF-1) and its receptor CXCR4. CXCR4 activation triggers a complex intracellular signaling including an increase in intracellular Ca^2+^ concentration whose role is still unclear. Using pharmacological and genetic approaches, we revealed that STIM1 and Orai1 were responsible for Ca^2+^ influx induced by SDF-1. Furthermore, we provide in vitro and in vivo evidence that they are necessary for basal or SDF-1-induced DLBCL cell migration which is independent of Ca^2+^ entry. We identify that they act as effectors coupling RhoA and ROCK dependent signaling pathway to MLC2 phosphorylation and actin polymerization. Finally, we revealed an alteration of Orai1 and STIM1 expression in extra-nodal DLBCL. Thus, we discovered a novel Ca^2+^-independent but Orai1 and STIM1-dependent signaling pathway involved in basal and CXCR4 dependent cell migration, which could be relevant for DLBCL physiopathology.

## 1. Introduction

Diffuse large B cell lymphoma (DLBCL) is the most common and one of the most aggressive types of non-Hodgkin lymphoma among adults. While nodal DLBCL typically develops in lymphatic organs, 30–40% of DLBCL arise at extra-nodal sites [1]. Differences in clinical presentation, molecular pathogenesis, and predisposing factors, indicate that extra-nodal DLBCLs are distinct entities [2]. One characteristic feature of nodal DLBCL is the dissemination of malignant B cells towards the organism but very little is known regarding the factors involved in their migration and trafficking [3]. The ability of B-cell lymphomas to spread to multiple organs reflects the migratory capacity of their normal counterparts. Indeed, B cells circulate continuously throughout the body via the blood and lymphatic systems. This trafficking is not random, but is under the control of chemokines such as Stromal Derived Factor 1 (SDF-1 or CXCL12) and its receptor CXCR4 [4]. Indeed, SDF-1-CXCR4 signaling plays a critical role in a variety of processes underlying proper B cell development including retention of precursor B cells in the bone marrow [5], homing of mature B cells in secondary lymphoid organs [6], trafficking and homing of plasma cell to bone marrow [7]. Moreover, it is now clearly established that SDF-1 is involved in DLBCL cell dissemination [8]. The binding of SDF-1 to CXCR4 initiates numerous intracellular signal transduction pathways which could be tissue-dependent and thus may differ between cell types, resulting in a variety of cellular responses such as chemotaxis, cell survival and proliferation, and gene transcription [9]. More precisely, upon activation, intracellular heterotrimeric G protein coupled to CXCR4 is dissociated into βγ dimer and αi monomer which subsequently activate the PLC/MAPK, PI3K/FAK and Rac/RhoA/Cdc42 pathways leading to the cell migration [10]. Furthermore, in hematopoietic progenitor cells and, more recently, in human endothelial progenitor cells, SDF-1 was shown to induce an increase in intracellular calcium concentration [Ca^2+^]_i_ necessary for cell migration [11,12] but whether this mechanism also occurs in B lymphoma cells is still unknown.

Until now, cell migration is considered as a Ca^2+^ dependent process given that several key molecular components and signaling events of the cellular migration are Ca^2+^ sensitive. Consequently, a fine distribution of intracellular Ca^2+^ gradient is needed to control directional migration and to orchestrate this intracellular distribution, Ca^2+^ channels are major regulators of this process [13]. In non-excitable cells, calcium entry is mainly due to store operated calcium entry (SOCE). By definition, SOCE is activated by endoplasmic reticulum (ER) Ca^2+^ store release. Two classes of proteins are mainly responsible for SOCE activity: STIMs (Stromal interaction molecule 1 and 2), ER Ca^2+^ sensors that detect ER Ca^2+^ store depletion, and Orais (Orai1, 2 and 3), the pore-forming subunits of plasma membrane Ca^2+^ channels. The Ca^2+^ release-activated Ca^2+^ (CRAC) channel, the archetype of store operated channel (SOC), is constituted of Orai1 and STIM1 and is the main Ca^2+^ channel responsible for Ca^2+^ entry in lymphocytes [14,15,16]. Both Orai1 and STIM1 have been described to be involved in the mediation of actomyosin assembly and the focal adhesions for migration of cancer cells [17,18,19]. However, all these studies have been performed on adherent cancer cells migrating in a mesenchymal manner while lymphocytes display a peculiar amoeboid type of migration [20] involving different molecular processes [3]. 

In the present study, we examined the role of Orai1 and STIM1 on basal and SDF-1-induced cell migration in DLCBL. We revealed that STIM1 and Orai1 mediate SOCE in response to SDF-1. However, we provided evidence, both in vitro and in vivo, that Orai1 and STIM1 are necessary for DLBCL migration but independently of Ca^2+^ entry across the plasma membrane. Our results suggest that STIM1 and Orai1 are required for RhoA/ROCK activation and MLC phosphorylation. Together, these data highlight a new role of STIM1 and Orai1 in lymphocyte migration.

## 2. Results

### 2.1. SDF-1 Induces Ca^2+^ Responses Involving Orai1/STIM1-Dependent Ca^2+^ Entry

**Figure 1:**
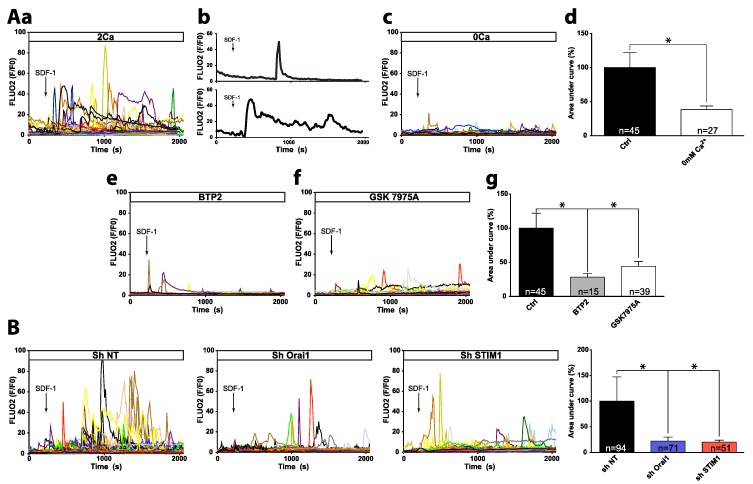
Stromal Derived Factor 1 (SDF-1) provokes an intracellular Ca^2+^ response in the HLY-1 diffuse large B cell lymphoma (DLBCL) cell line involving intracellular Ca^2+^ pool mobilization and Orai1/STIM1 extracellular Ca^2+^ influx. Ca^2+^ responses to SDF-1 (100 ng/mL) were measured using Fluo2-LR-AM Ca^2+^ dye and recorded by videomicroscopy (Zeiss LSM 510) using ×25 objective. Black arrows indicate SDF-1 addition. Each trace represents the response of one cell and data are representative of at least three independent experiments. Typical response of unique cell (peak or peak follow by sustained plateau phase) are present as example (**Ab**). Data were processed using GraphPad prism. (**A**) Pharmacological characterization of SDF-1-induced Ca^2+^ increase. Cells were recorded in extracellular saline solution (HBSS) containing 2 mM Ca^2+^ (2 Ca, (**Aa**)) or in Ca^2+^-free HBSS (0 Ca, (**Ac**)). Cells were pre-incubated with BTP2 (**Ae**) or GSK7975A (**Af**) at 10 µM for 30 min and recorded in 2 mM Ca^2+^ HBSS containing inhibitors. (**Ad**,**Ag**) Histograms represent areas under curves (AUC) calculated, under various recording conditions, between the application time of SDF-1 and t = 2050 s, and normalized compared to control (2 Ca or shNT). Data are expressed as mean ± SEM, * *p* < 0.05. (**B**) Effect of Orai1 or STIM1 expression knock-down on SDF-1-induced Ca^2+^ response. The stable modified HLY-1 cell line established after lentiviral transduction with plasmid containing non targeting shRNA (shNT), shRNA against Orai1 or STIM1 were recorded in extracellular saline solution (HBSS) containing 2 mM Ca^2+^.

The addition of SDF-1 triggered a cytosolic Ca^2+^ increase in SU-DHL-4 and HLY-1 cell lines, consisting of peaks and/or a sustained plateau phase (Figure 1(Aa,b) and Figure S1(Aa,b)). To determine the origin of these Ca^2+^ responses, cells were recorded in Ca^2+^-free medium (Figure 1(Ac) and Figure S1(Ac)). Ca^2+^ responses were maintained, but the areas under the curves were statistically smaller in both cell types (Figure 1(Ad) and Figure S1(Ad)), suggesting that these responses resulted from both intracellular Ca^2+^ store mobilization and extracellular Ca^2+^ influx. To determine whether CRAC channels participated in Ca^2+^ influx triggered by SDF-1, pharmacological and RNAi approaches were used. To evaluate and validate the efficacy of the CRAC channel blockers and shRNAs targeting Orai1 and STIM1 on SOCE, we used the most common means to activate SOCE by inducing ER Ca^2+^ depletion using the sarcoplasmic/endoplasmic reticulum Ca^2+^-ATPase (SERCA) inhibitor thapsigargin [21]. Treatment of DLBCL cell lines with thapsigargin in Ca^2+^-free medium led to a transient response corresponding to Ca^2+^ release from ER-Ca^2+^ stores (Figure S2). Addition of 2 mM Ca^2+^ to the recording medium revealed a second response due to Ca^2+^ entry through SOCE. Thus, we revealed that BTP2 and GSK7975A, two CRAC channel blockers, inhibited more than 80% of SOCE in both cell lines (Figure S2A) confirming their high efficacy to block SOCE. In the same way, we validate the efficiency of shRNA Orai1 and STIM1 which significantly reduced SOCE-induced by thapsigargin as well as Orai1 and STIM1 protein expression, respectively (Figure S2C). To note that the kinetic of the initial rise of the residual SOCE was not altered, as commonly observed [22,23], in down-expressing Orai1 and STIM1 SU-DHL-4 cells suggesting that beside Orai1, another SOC like TRPC1, could contribute to SOCE in these cells.

When cells were pretreated with BTP2 or GSK7975A, they exhibited significantly lower SDF-1-induced Ca^2+^ responses (Figure 1(Ae–g) and Figure S1(Ae–g)). Similarly, Ca^2+^ responses induced by SDF-1 were significantly attenuated in Orai1 or STIM1 knockdown cells compared to cells expressing a non-targeting shRNA (shNT) (Figure 1B and Figure S1B). These results suggest that SDF-1 provoked an increase in [Ca^2+^]_i_, involving the mobilization of intracellular Ca^2+^ stores and the activation of an extracellular Ca^2+^ influx originating from Orai1/STIM1 CRAC channels. To determine whether the CXCR4/SDF-1 axis was responsible for the [Ca^2+^]_i_ increase, cells were pretreated with AMD3100, a CXCR4 inhibitor. We observed that Ca^2+^ response to SDF-1 was significantly impaired in AMD3100-treated cells (Figure S3A), suggesting that SDF-1-induced Ca^2+^ response is mainly mediated by CXCR4 in both cell lines.

### 2.2. Calcium Independent Involvement of Orai1 and STIM1 in DLBCL Migration

**Figure 2:**
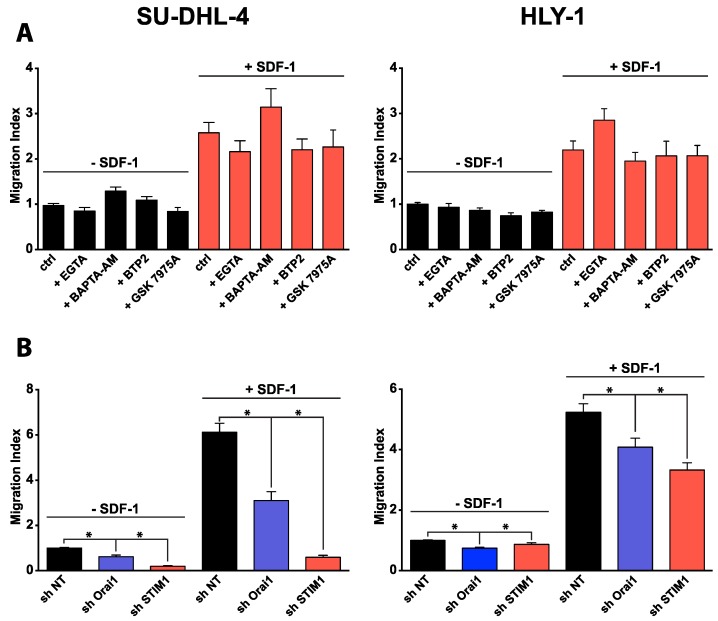
Orai1 and STIM1 regulate basal and SDF-1-induced DLBCL cell migration in a Ca^2+^ independent manner in vitro. Cell migration was assessed in 96-transwell chemotaxis chambers assay. Histograms represent mean ± SEM from at least 3 independent experiments, * *p* < 0.05. (**A**) Ca^2+^ is not necessary for DLBCL cell migration. To test the effect of the pharmacological agents on chemotaxis induced by SDF-1 (100 ng/mL), cells were pre-treated during 20 min in the presence or not of the agents before to be loaded to upper transwell chambers and pharmacological agents were maintained in medium during the experiment. BAPTA-AM, intracellular Ca^2+^ chelator, 5 µM; EGTA, extracellular Ca^2+^ chelator, 1 mM; BTP2 and GSK7975A, CRAC inhibitors, 10 µM. (**B**) Orai1 and STIM1 are required for DLBCL migration. Basal and SDF-1 (100 ng/mL)-induced migration were measured (as described above) in stable modified HLY-1 and SU-DHL-4 cells established after lentiviral transduction with plasmid containing non targeting shRNA (shNT), shRNA against Orai1 or STIM1.

It is well known that SDF-1 is a potent chemoattractant for DLBCL cells. However, the role of Ca^2+^ in the pro-migratory effect of SDF-1 remains unclear. We performed pharmacological and RNA interference analyses to address this question. First, using transwell assays, we evaluated the chemotactic effect of SDF-1 in SU-DHL-4 and HLY-1 cell lines. As expected, we observed that SDF-1-induced migration in both cell lines was completely abolished in the presence of AMD3100 (Figure S3B). These results suggest that SDF-1 stimulate DLBCL migration via an action mechanism involving CXCR4. We then investigated the role of Ca^2+^ in SDF-1 pro-migratory effect. Surprisingly, pre-treatment of cells with extracellular (EGTA) or intracellular (BAPTA-AM, Figure S2B) Ca^2+^ chelator, or CRAC inhibitors (BTP2, GSK7975A) had no effect on basal and SDF-1-induced migration in either cell line (Figure 2A). However, we show that the down-regulation of STIM1 and Orai1 expression significantly altered the basal and SDF-1-induced migration of SU-DHL-4 and HLY-1 cells. Indeed, the basal and SDF-1-induced migration was drastically or partly inhibited in shSTIM1 and shOrai1-expressing SU-DHL-4 cells, respectively (Figure 2B). To a lesser extent, similar effects were obtained in HLY-1 cells under-expressing Orai1 and STIM1 (Figure 2B). Weaker effects observed in HLY-1 than in SU-DHL-4 cells may be due to a lower efficacy of shRNA in HLY-1 than in SU-DHL-4 cells (Figure S2C). Finally, we checked that the knockdown of Orai1 and STIM1 had no effect on basal total and membrane CXCR4 expression (Figure S3C,D). These results show that DLBCL cell migration required Orai1 and STIM1 but not Ca^2+^ signaling, suggesting a new Ca^2+^-independent role of Orai1/STIM1 in malignant B lymphocytes.

### 2.3. STIM1 Knock-Down Impaired DLBCL Dissemination In Vivo

**Figure 3:**
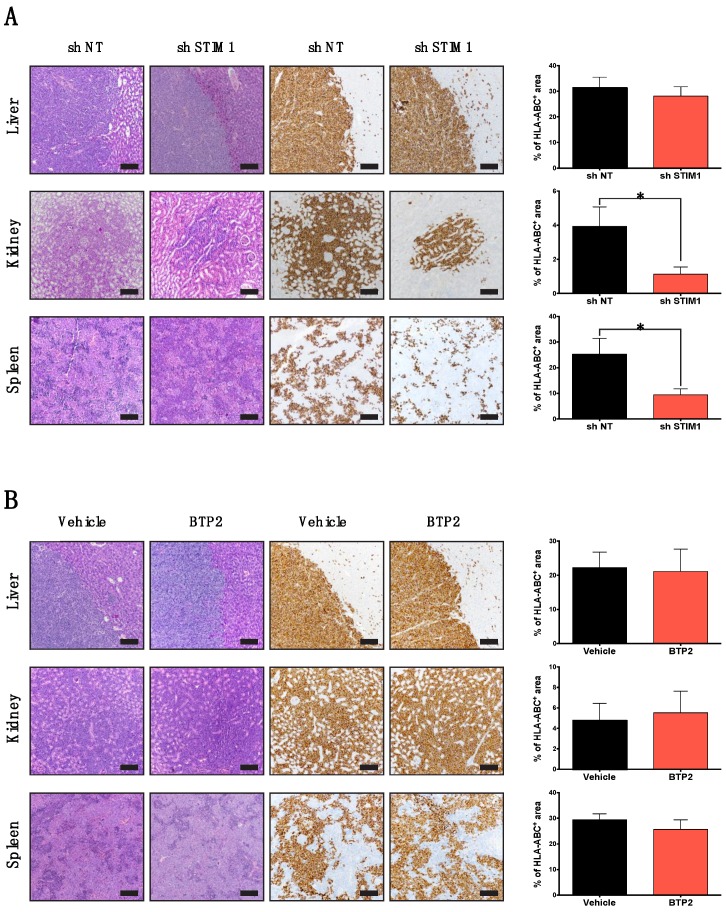
STIM1, but not Ca^2+^, regulate DLBCL dissemination in vivo. (**A**) Effect of STIM1 under-expression on HLY-1 cell dissemination. (**B**) Effect of intraperitoneal injection of BTP2 (12 µg/kg) or vehicle, three times per week on HLY-1 cell dissemination. Images were captured with a Nikon Eclipse Ci microscope equipped with a Plan Fluor 10× 0.3 NA objective. Scale bars = 150 μm. Histograms represent the quantification of positive surface for HLA-ABC staining. All tissues were delimited and to evaluate the percentage of positive surface for HLA-ABC staining on tissue, thresholding on positive and negative staining was done using Mercator software. Data are represented as mean ± SEM (n = 10), * *p* < 0.05.

To test the role of CRAC channels in DLBCL dissemination, mice were intra-hepatically xenografted [24] with HLY-1 cells expressing shNT or shSTIM1. We chose these experimental conditions due to the fact that (1) only the HLY-1 cell line has the ability to induce an intra-hepatic tumor and spread into organs and (2) shSTIM1 was the most efficient to inhibit cell migration in vitro compared to shOrai1. Four weeks after engraftment, mice were sacrificed and necropsy was performed. Liver, spleen, and kidneys were systematically removed and invasion of these organs by human tumor cells was evaluated by immunostaining for the human cell marker HLA-ABC. Firstly, we checked that the under-expression of STIM1 was maintained in vivo by performing immunofluorescence on the liver tumor (Figure S4A). We then analyzed, by immunohistochemistry (IHC), the labelling of HLA-ABC in injected livers. As shown in Figure 3A, regardless of the type of cells injected, the percentage of HLA-ABC+ tumor cells in the liver at the end of the experiment was similar. This suggests that there is no difference in the ability of cell engraftment and proliferation based on the expression of shSTIM1 or shNT, as confirmed by quantification of Ki67 labelling in injected livers (Figure S4B) and corroborating in vitro data revealing no difference in cell growth between shNT and shSTIM1 expressing cells (Figure S4C). In comparison to control cells, STIM1 knockdown tumor cells had reduced capacity to colonize organs such as spleen and kidneys (Figure 3A). The evaluation of the percentage of HLA-ABC+ cells by flow cytometry showed that spleen and kidneys were invaded by two-fold more tumor cells in mice xenografted with HLY-1 shNT cells than shSTIM1 cells (Figure S4C). To confirm the Ca^2+^-independent role of STIM1 in DLBCL cell dissemination in vivo, mice were intra-hepatically xenografted with HLY-1 cells and treated three times per week with BTP2 or vehicle for four weeks [25]. Analyses of tumors by IHC or flow cytometry clearly revealed that there was no difference between mice treated with BTP2 or not (Figure 3B and Figure S4D). Taken together, these observations suggest that STIM1 is likely involved in DLBCL dissemination and that it acts through a Ca^2+^-independent mechanism.

### 2.4. Molecular Mechanisms Mediating Orai1/STIM1 Dependent DLBCL Migration

**Figure 4:**
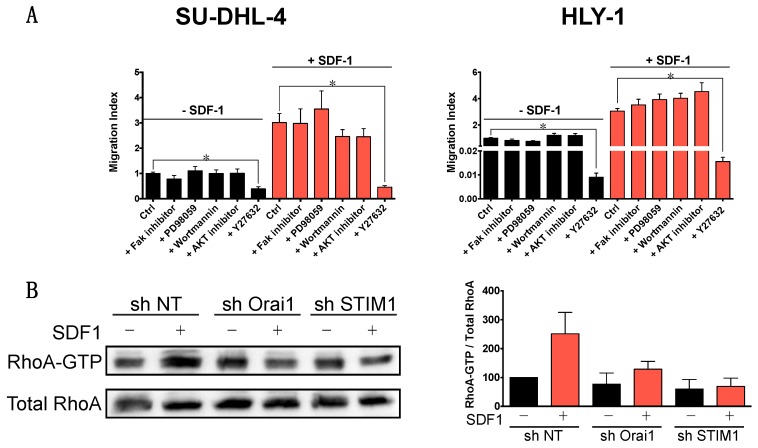

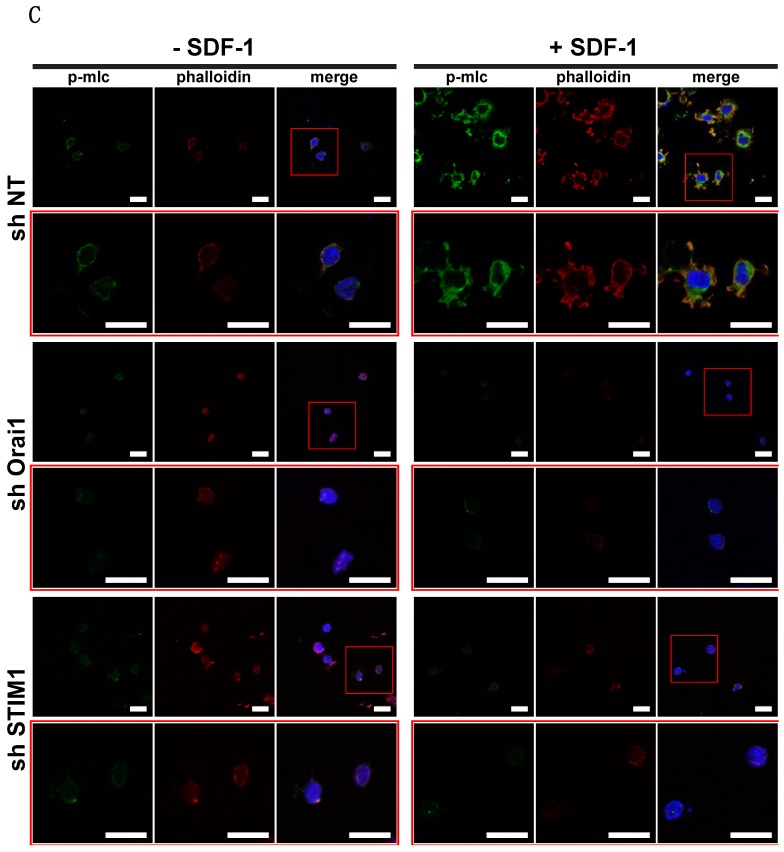
Orai1 and STIM1 control DLBCL cell migration through RhoA activation, ROCK, and MLC phosphorylation. (**A**) SDF-1-induced SU-DHL-4 and HLY-1 cell migration is ROCK activation dependent. Cells incubated or not with SDF-1 (100 ng/mL) were pre-treated during 20 min in the presence or not of various inhibitors (FAK inhibitor 1 µM, PD98059 a MEK inhibitor 10 µM, Wortmannin a PI3K inhibitor 10 nM, AKT inhibitor 250 nM, Y27632 a ROCK inhibitor 1 µM). Data are represented as mean ± SEM of 3 independent experiments, * *p* < 0.05. (**B**) SDF-1-induced RhoA activation is impaired by shOrai1 and shSTIM1 in SU-DHL-4 cells. *Left*: Cells were treated for 30 min with SDF-1 (200 ng/mL) and RhoA activation was evaluated by pull-down assay and western blot. *Right*: Quantification of western blot performed after pull-down assays (n = 3) (**C**) Orai1 and STIM1 act as regulators of the MLC phosphorylation. Immunofluorescence of SU-DHL-4 cells under-expressing, or not, Orai1 or STIM1 seeded on glass coverslips, fibronectin coated, were stimulated, or not, with SDF-1 (200 ng/mL) for 1 h. Ser^19^phosphoMLC2 was immunostained with mouse anti-phosphoMLC2 mAb revealed using secondary Alexa488-coupled donkey anti-mouse Ab (in green), F-actin was revealed with Phalloïdin-AlexaFluor 594 (in red) and nuclei were stained using Hoechst 33258 (in blue). Cells in square are zoomed and shown below. Images were acquired using a Zeiss LSM 510 meta confocal microscope (Zeiss, Göttingen, Germany) with an ApoPLAN 63× objective. Scale bar = 20 µm.

SDF-1 has been shown to initiate cell chemotaxis by activation of various transduction pathways which are dependent on cell types [9] and microenvironment [26]. To determine which transduction pathway was involved in the Ca^2+^-independent effect of Orai1 and STIM1 on basal and SDF-1-induced DLBCL cell migration, we tested the effect of inhibitors targeting the main kinases previously described as involved in cell migration [9]. A specific abrogation of basal and SDF-1-induced cell migration was observed in SU-DHL-4 and HLY-1 cells in the presence of the ROCK inhibitor (Y-27632) while the other inhibitors (FAK inhibitor, PD98059 a MEK inhibitor, Wortmannin a PI3K inhibitor and AKT inhibitor), had no effect (Figure 4A). As ROCK is activated by RhoA, we therefore evaluated the activation of RhoA in cells under-expressing STIM1 or Orai1 using a pull-down assay. Our results show that RhoA activation by SDF-1 was impaired in cells under-expressing STIM1 or Orai1 suggesting a major role of the RhoA/ROCK pathway in SDF-1-induced B lymphocyte migration (Figure 4B). Finally, using immunofluorescence and phalloïdin stainings, we clearly show that SDF-1 induced the phosphorylation of MLC2 and actin rearrangement in SU-DHL-4 cells after 1 h of treatment, but this effect was completely inhibited in cells expressing shOrai1 or shSTIM1 (Figure 4C). These results indicate that Orai1 and STIM1 act on DLBCL cell migration as effectors coupling the SDF-1 receptor to RhoA/ROCK/MLC2 pathway.

### 2.5. STIM1 and Orai1 Expression in Diffuse Large B Cell Lymphomas

**Figure 5:**
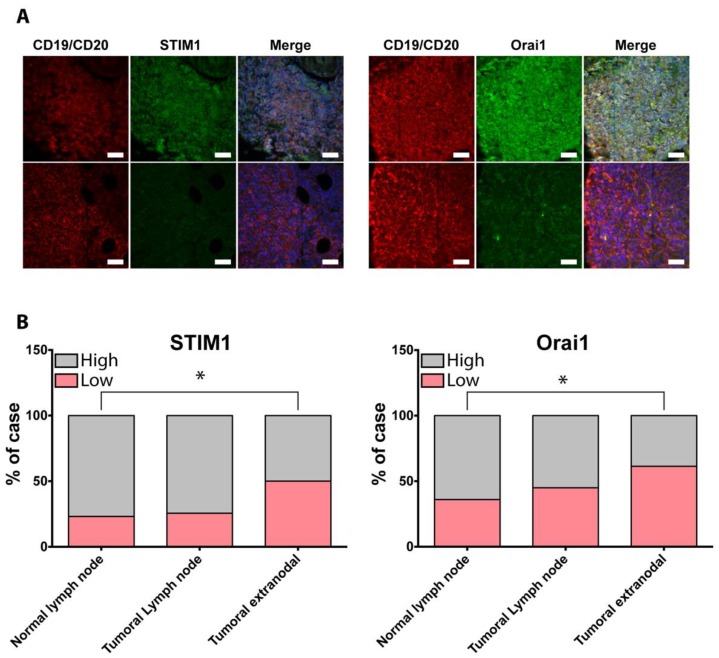
Orai1 and STIM1 expression is altered in extra-nodal DLBCL. (**A**) Representative immunofluorescent staining of STIM1 and Orai1 in normal lymph node and extra-nodal DLBCL under-expressing STIM1 or Orai1. Images were acquired using Leica DMI8 microscope equipped with a 40× oil immersion objective. TMA including samples from normal lymph node (n = 26), nodal (n = 43) and extra-nodal (n = 44) DLBCL was co-immunostained using Orai1 or STIM1 antibodies revealed by donkey anti-rabbit Alexa-488 (in green) and mouse anti-human CD20 and anti-human CD19 revealed by goat anti-mouse Alexa 532 (in red). Nuclei were stained with DAPI (in blue). Scale bar = 50 µm. (**B**) Quantification of STIM1 or Orai1 alteration in DLBCL samples. After acquisition of spot fluorescence on Icys laser scanning cytometer, a segmentation analysis base on phantoms was done to determine the percentage of CD20/CD19 positive cells expressing protein of interest for each spot. The stacked column charts illustrate the intensity grade for STIM1 or Orai1 expression in surgical specimens. * *p* < 0.05 by Chi-square test.

To study the clinical relevance of Orai1 and STIM1 in DLBCL dissemination, their expression was examined in 26 normal lymph nodes and 87 tumor tissues from nodal (n = 43) and extra-nodal (n = 44) DLBCL surgical samples. Thus, commercial TMA were co-immunostained for Orai1 or STIM1 and CD19/CD20 (Figure 5A), and fluorescence was analyzed using laser scanning cytometry technology [20]. To quantify Orai1 and STIM1 expression, we scored the samples by the distribution and intensity of immunofluorescent staining for Orai1 or STIM1 in B cells (CD19 and/or CD20 positive cells). In normal tissue, 77% of cases (20/26) exhibited high score for STIM1 expression and 23% (6/26) showed low score. Similar results were obtained in nodal DLBCL. However, in extra-nodal DLBCL, the repartition significantly differed from normal lymph node with 50% of cases (15/30) showing a low grade of STIM1 expression (*p* < 0.05 compared to the intensity grades for STIM1 in normal tissue) (Figure 5B). Similarly, we observed that high grades of Orai1 expression are in the majority in normal lymph nodes and nodal DLBCL (16/25 = 65% in normal tissue vs. 22/48 = 55% in nodal DLBCL). In contrast, the majority of extra-nodal DLBCL cases exhibited low grade intensity of Orai1 expression (27/44 = 61% of low grade in extra-nodal DLBCL vs. 9/25 = 36% in normal tissue, *p* < 0.05) (Figure 5B). These results show that the expression of Orai1 and STIM1 is lower in extra-nodal DLBCL compared to normal tissue and nodal DLBCL.

## 3. Discussion

In the present work, we highlight a novel, Ca^2+^-independent role of Orai1 and STIM1 in basal and SDF-1-induced DLBCL cell migration. We confirm that SDF-1 induces an intracellular Ca^2+^ increase and demonstrate that Orai1 and STIM1 are responsible for the extracellular Ca^2+^ influx induced by SDF-1. Moreover, we clearly demonstrate, both in vitro and in vivo, that basal or SDF-1-induced B-cell migration is dependent on STIM1 and Orai1 expression independent of intracellular Ca^2+^ increase. We identify that STIM1 and Orai1 act as effectors coupling RhoA/ROCK to MLC phosphorylation leading to actin polymerization. Finally, clinical sample analyses showed a decrease in Orai1 and STIM1 expression in a significant proportion of extra-nodal DLBCL which could have an impact on their clinical presentation and evolution.

During their life, normal and tumor B lymphocytes circulate around the body via the lymphatic system and blood. Their migration is mainly regulated by chemokines such as SDF-1. The various transduction pathways activated by the SDF-1/CXCR4 axis have been extensively studied in adherent normal and cancer cells. They include activation of heteromeric G-proteins which mainly induce PI3K, PLC, and Rho/Rac/Cdc42 as downstream effectors resulting in a variety of cellular responses [9]. Consequent to the PLC activation, SDF-1 triggers an intracellular Ca^2+^ concentration increase involving IP3-dependent Ca^2+^ pool mobilization and extracellular Ca^2+^ influx [12,27]. We confirm these observations in DLBCL cell lines and reveal, using pharmacological and expression knockdown approaches, that Orai1/STIM1 channels are major molecular actors of SDF-1-induced extracellular Ca^2+^ influx. By evoking Ca^2+^ signals and other signaling pathways, SDF-1 may influence many cellular processes including chemotaxis. However, our results demonstrate that extra (EGTA)- or intracellular (BAPTA-AM) Ca^2+^ chelator inhibiting Ca^2+^ release and Ca^2+^ entry signal in response to TG or SDF-1, as well as specific Orai1/STIM1 Ca^2+^ channel inhibitors, had no effect on basal or SDF-1-induced migration in tumor B cells which strongly suggest that intracellular Ca^2+^ increase is not required for B cell migration. The role of Ca^2+^ in mesenchymal cell migration has been widely demonstrated [28], while in amoeboid migration, used by lymphocytes, it is more controversial. Indeed, in T lymphocytes, intracellular Ca^2+^ increase was reported to inhibit T-cell motility and might act as a “stop signal” [29,30]. More specifically, in B lymphocytes, it has been shown that migratory response to chemokines such as SDF-1 could be Ca^2+^-independent according to the B cell maturation stage [31,32]. So, SDF-1-induced Ca^2+^ responses in tumor B cells are likely involved in other processes such as cell survival or transcription gene expression. More intriguing is the fact that the under-expression of Orai1 and STIM1 resulted in a drastic decrease of DLBCL migration. These results have been corroborated by in vivo experiments using a mouse model of DLBCL dissemination from an intrahepatic xenograft to various organs [24] which showed that the inhibition of the Ca^2+^ channel activity of Orai1/STIM1 by BTP2 was inefficient to impair B cell dissemination in contrast to STIM1 under-expression.

Similar non-conductive roles for channel proteins have been described previously. More specifically, it has been shown that Orai1 and Orai3 proteins were more important than calcium influx to control cell proliferation in various solid cancer cell lines [33], and there is mounting evidence for Ca^2+^ independent effect of TRP channels on gene expression, DNA damage, cytoskeletal dynamics, and migration [34,35]. Our results are also consistent with previous studies showing that thrombin-mediated disruption of endothelial barrier required STIM1 but was independent of Ca^2+^ entry across the plasma membrane [22,36]. As these studies revealed that STIM1 acted independently from Orai1 in endothelial cells, we noted that despite a lower effect of shSTIM1 than shOrai1 on SOCE, the downregulation of STIM1 seems more efficient to inhibit cell migration than that of Orai1. This leads to the hypothesis that, according to the process studied, STIM1 might act as a driver molecule involved in various Ca^2+^-independent processes while Orai1 could be a passenger molecule. Further experiments will be necessary to highlight this point.

Previously, the RhoA/ROCK/MLC transduction pathway has been identified as a mechanism for the STIM1-mediated contribution to thrombin-induced disruption of endothelial barrier. Interestingly, our data reveal a similar pathway to link the Ca^2+^-independent effect of Orai1 and STIM1 to SDF-1-induced migration in DLBCL cell lines. Indeed, we clearly demonstrate that inhibition of ROCK, a key downstream effector of RhoA, drastically blocks basal and stimulated DLBCL cell migration. Although the precise involvement of the RhoA/ROCK pathway in lymphocyte biology has not been fully elucidated, the best described role of this pathway seems to be the control of cytoskeletal reorganization [37]. We clearly demonstrate that Orai1 and STIM1 participated in this pathway since their knockdown prevented the phosphorylation of MLC2 and consequently the polymerization of actin involved in the lymphocyte migration [38]. Altogether, these observations reveal a new, Ca^2+^-independent, role of Orai1 and STIM1 in DLBCL cell migration. More importantly, our results strengthen the data obtained by the Trebak’s group [22,36] and highlight the putative existence of a novel RhoA/ROCK/MLC transduction pathway, involving Orai1 and/or STIM1 as Ca^2+^-independent effectors, which could be activated in various cellular contexts requiring cytoskeletal reorganization.

Although Orai1 and STIM1 are the major components of CRAC channels, many other auxiliary proteins have been described to interact and regulate Orai1/STIM1 functions [39,40]. Thus, we assume that the Ca^2+^ independent effect of Orai1/STIM1 on DLBCL cell migration could be mediated by protein–protein interactions, as previously described between TRPM8 channel and the small GTPase Rap1 causing endothelial cell migration inhibition [35]. More specifically, as previously demonstrated by Chen et al. in solid cancer cells [18], we could hypothesize that after stimulation, the microtubule plus-end binding protein EB1 binds STIM1, linking it with microtubules and finally activating RhoA/ROCK-dependent cell migration [41]. Nevertheless, further experiments are needed to elucidate the exact molecular mechanism by which Orai1 and STIM1 interfere with DLBCL cell migration.

Alterations of Orai1 and even more STIM1 in solid cancers have been exhaustively studied [19] but, to our knowledge, no data are available concerning the expression of these proteins in hematologic malignancies. Our clinical samples study indicates that Orai1 and STIM1 are under-expressed in half of the extra-nodal DLBCL while no significant modification was observed in lymph node DLBCL compared to normal lymph node. Although surprising, these results could account for specific properties of extra-nodal DLBCL which exhibit an initial confinement to a single anatomical site and less propensity to disseminate [42]. Interestingly, a recent study has shown that, in contrast to solid cancers, intra-lymphatic spread of extra-nodal DLBCL lymphoma cells was a rare event [43] which might explain the lesser propensity of these tumors to disseminate. Thus, the down-expression of STIM1 or Orai1 could impair cell migration and/or intralymphatic spread, promoting homing of tumor B cells to extra-nodal sites. Further experiments should be necessary to confirm this hypothesis.

## 4. Materials and Methods

### 4.1. Reagents and Antibodies

Fibronectin, EGTA, and BAPTA-AM were purchased from Sigma-Aldrich (L’Isle d’Abeau, France). BTP2 was from Interchim (Montluçon, France). GSK7975A was supplied by Aobious (Gloucester, MA, USA). SDF-1 was from Peprotech (Neuilly sur seine, France). Indo-1-AM and Alexa fluor 594 Phalloidin were from Life Technologies (Courtaboeuf, France). Puromycin, AMD 3100, and Y27632 were supplied by Tocris (Bio-Techne, Lille, France). Fluo-2-AM-LR was from Teflabs and Rho Activation Assay Biochem Kit was supplied by Cytoskeleton (Euromedex, Mundolsheim, France). Mouse anti-human CD19 were supplied by Diagomics (Blagnac, France), mouse anti-human CD20 and monoclonal mouse anti-human Ki67 clone MIB-1 were from Agilent Technologies (Les Ulis, France). Alexa532-conjugated goat anti-mouse and Alexa 488-conjugated donkey anti-rabbit polyclonal antibodies came from Life Technologies (Saint-Aubin, France). The mouse anti-human HLA-ABC–PECy7 clone G46-2.6 and rat anti mouse CD16/CD32 were from BD biosciences (Le Pont de Claix, France). PE/Cy5 anti-human CXCR4 (clone 12G5) and PE/Cy5 mouse IgG2α, κ isotype control were supplied by BioLegend (Ozyme). Western blots were performed using anti-human Orai1 or STIM1 (Alomone labs, Jerusalem, Israël), anti-CXCR4 (Santa Cruz Biotechnology, Heidelberg, Germany) rabbit polyclonal antibodies, mouse anti-human βactin (Sigma Aldrich, L’Isle d’Abeau, France), and anti-phosphoMLC2 (Cell Signaling Technology, Ozyme, Saint Quentin en Yvelines, France) monoclonal antibodies.

### 4.2. Cell Lines

SU-DHL-4 cell line was obtained from the Deutsche Sammlung von Mikrooganismen und Zellkulturen GmbH cell collection (Braunschweig, Germany). HLY-1 cell line was generously provided by Dr. Fabienne Meggetto, Toulouse, France. Cells were cultured in RPMI-1640 media supplemented with 10% FBS under humid atmosphere containing 5% CO_2_. Stable modified cell lines SU-DHL-4 and HLY-1, were established after transduction with lentivirus carrying the PLKO1.5 plasmid containing shRNA against STIM1 (Sigma TRCN0000149588) or Orai1 (Sigma TRCN0000165044) as previously described [25]. The pXS68 non targeting shRNA (shNT) was used as lentiviral transduction control. The transduced cells were selected by treating them with Puromycin (1 µg/mL for SU-DHL-4 and 2.5 µg/mL for HLY-1).

### 4.3. Calcium Imaging

Single-cell [Ca^2+^]_i_ imaging was performed, using Fluo2-AM-leak resistant (LR) calcium dye. Cells were loaded with 10 µM Fluo2-AM-LR in the presence of 0.02% pluronic F127 at room temperature in HBSS for 25 min. The cells were rinsed with HBSS and incubated in the absence of Fluo2-AM-LR for 15 min to allow complete de-esterification of the dye. Fluorescence time-lapse images were captured at 515 nm using a confocal microscope Zeiss LSM 510 (Göttingen, Germany) equipped with a Plan Neofluar 25× 0.8 NA oil objective. The recording lasted 2050 s and SDF-1 was added at t = 200 s. Images were recorded at constant 10 s intervals under the control of LSM 510 software (Zeiss). Regions of interest corresponding to recorded cells were drawn to analyze fluorescence signal. Data were processed using Prism 6 (GraphPad).

In some experiments, cells were placed in a Ca^2+^-free medium consisting of the HBSS in which CaCl_2_ was omitted and 100 µM EGTA was added in order to chelate residual Ca^2+^ ions. This medium was added to the cells just before recording to avoid leak of the intracellular calcium stores. Each experimental condition was repeated independently at least three times. Area under curve was calculated from 200 s to 2050 s of the recording.

### 4.4. Transwell Assay

Transmigration of DLBCL cell lines was assessed in 96-transwell chemotaxis chambers with a pore size of 5 µm (Corning). Cells (5 × 10^5^) were rinsed and suspended in serum-free media before being loaded in the upper chamber of the transwell culture insert. The bottom chamber was filled with culture medium with or without SDF-1 (100 ng/mL). To test the effect of the pharmacological agents on chemotaxis induced by SDF-1, cells were pre-treated during 20 min in the presence, or not, of the agents before being loaded to upper transwell chambers. After 10 hours of incubation for SU-DHL-4 and 16 hours for HLY-1 cells, the upper chambers were removed and cells that had migrated were counted after adding an internal microsphere counting standard (Precision count beads, Ozyme) by flow cytometry using a FACSCalibur cytometer equipped with an HTS module and Cell Quest software (BD Biosciences). Results are presented as migration index which was calculated by dividing the number of migrated cells in the studied condition by the number of migrated cells in the absence of chemoattractant (control).

### 4.5. In Vivo Experiments

Animal experiments were performed in A2 animal facility (Bordeaux University), in accordance with national institutional guidelines and with the agreement of the local Ethic Committee on Animal Experiments CEEA50 of Bordeaux (2903-2016051011358065). To assess the cell spreading into organs, intrahepatic xenografts were performed as previously described [24]. Briefly, 5 × 10^5^ HLY1 cells expressing shNT or shSTIM1 were intrahepatically xenografted in 8 to 15 weeks old NSG immunodeficient mice (NOD.Cg-Prkdcscid Il2rgtm1Wjl/SzJ) under 2.5% isoflurane anesthesia (Belamont, Piramal Healthcare, Northumberland, UK). For pharmacological treatments, HLY-1 parental cells were intrahepatically xenografted and mice were treated by intraperitoneal injection of vehicle or SOCE inhibitor BTP2 (12 µg/kg) three times per week [25]. A global surveillance of animal health was performed two to three times per week. Four weeks after engraftment, mice were sacrificed. Liver, spleen, and kidneys were removed and cut in half. One half was fixed in 4% formaldehyde for immunohistochemistry and the other half was kept for flow cytometry analysis. Ten mice were grafted for each cell line.

### 4.6. RhoA Activation Assay

RhoA activity was measured using cytoskeleton RhoA activation assay kit according to the manufacturer’s recommendations. Briefly, cells were lysed in 700 μL of lysis buffer. Lysates (800 μg) were cleared at 15,000× *g* and the supernatants were rotated for 1 h with 50 μg of Rhotekin-RBD Beads. Samples were then washed three times and immunoblotted with RhoA monoclonal antibodies. Whole cell lysates were also immunoblotted for RhoA as loading controls.

### 4.7. Immunohistochemistry

To analyse liver engraftment and human lymphoma dissemination in organs harvested, H&E staining and human leucocyte antigen (HLA-ABC) IHC were performed on formalin-fixed paraffin-embedded mice organ sections (3 µm thick). HLA-ABC expression was revealed using mouse anti-human HLA-ABC clone G46-2.6 antibody (1:3000) and ultraview universal DAB detection kit (Ventana, Roche, Basel, Switzerland). Slides were scanned using panoramic scan (3Dhistech, Sysmex, Villepinte, France) and then analyzed using Mercator software (Exploranova, La Rochelle, France). Briefly, tissue was delimited and thresholds for positive and negative staining were determined to evaluate the percentage of positive area for HLA-ABC staining on tissue. Images of H&E and HLA-ABC staining were acquired using a Nikon Eclipse Ci microscope equipped with a Plan Fluors 10× 0.3 NA objective.

### 4.8. Flow Cytometry Analysis

HLA-ABC was detected in single cell suspensions from enzymatic and mechanical dissociated organs, using PE-Cy7 antibody (BD Biosciences) and analyzed by BD FACS CantoII flow cytometer and FlowJo software (Tree Star Inc., Ashland, OR, USA).

### 4.9. Tissue Microarrays

Tissue Microarrays (TMA) were purchased from US Biomax (Rockville, MD, USA). TMA were composed of 26 spots of normal lymph node and 87 spots of DLBCL tumoral tissue divided into 43 spots of nodal and 44 spots of extranodal DLBCL. Co-immunostainings were performed using Orai1 antibody (HPA016583, Sigma) or STIM1 antibody (HPA012123, Sigma) and mouse anti-human CD20 and anti-human CD19 revealed by Alexa 488-conjugated donkey anti-rabbit and Alexa532-conjugated goat anti-mouse polyclonal antibodies, respectively. Nuclei were stained with DAPI. After acquisition on Icys laser scanning cytometer (Thorlabs, Maison Lafitte, France), a segmentation analysis base on phantoms was done to determine the percentage of CD20/CD19 positive cells expressing protein of interest for each spot as described previously [44]. To quantify Orai1 and STIM1 expression, we graded the samples as follows: the high or low grade indicates that the mean percentage of B cells (CD20 and/or CD19 positive) expressing Orai1 or STIM1 is higher or lower than the mean observed in normal lymph node, respectively.

### 4.10. Western Blot

Cell lysates from various cell lines were prepared using Cell Signaling Technology cell lysis buffer supplemented with protease/phosphatase inhibitor cocktails (Cell Signaling Technology, Ozyme, Saint Quentin en Yvelines, France). Proteins were loaded onto 10% Tris-acrylamide gels, transferred with I-blot system (Thermofisher Scientific, Bordeaux, France) on PVDF membrane and blotted with anti-human Orai1 or STIM1 antibodies overnight at 4 °C. Proteins of interest were visualized using a chemiluminescent HRP substrate kit (Merck-Millipore, Fontenay sous bois, France) revealed with a Fusion system (Vilber Lourmat, Marne-laVallée, France) and analyzed using Bio1D software (Vilber Lourmat). The densitometry values were normalized with those of β-actin (loading control).

### 4.11. Confocal Microscopy

Cells were plated on glass coverslips pre-coated with fibronectin at 2 µg/cm^2^. Cells were rinsed twice in serum-free medium and treated with or without SDF1 for 1 h. After fixation in PBS containing 4% (*w/v*) paraformaldehyde for 10 min, cells were then permeabilized in PBS supplemented with 0.2% triton X-100 for 5 min. After a step of saturation with a solution containing PBS and 0.2% gelatin for 30 min, cells were incubated with anti-phosphoMLC2 mAb in PBS/0.2% gelatin overnight at 4 °C. Phospho-MLC was revealed using secondary Alexa488-coupled donkey anti-mouse Ab. F-actin and Nuclei were stained using phalloïdin and Hoechst 33258, respectively. Images were acquired using a Zeiss LSM 510 meta confocal microscope (Zeiss, Göttingen, Germany) with an ApoPLAN 63× objective.

### 4.12. Statistical Analysis

Data are shown as mean ± standard error of mean (SEM). The significance of differences was calculated using a 2-tailed unpaired Student t-test or Mann and Whitney or one-way ANOVA or chi-square test, as indicated. Differences were considered statistically significant when *p* < 0.05 (*).

## 5. Conclusions

We provide evidences that DLBCL migration is regulated by Orai1 and STIM1 expression but not by intracellular Ca^2+^ concentration, revealing by the way additional functions for these proteins. Further experiments should be done to determine the precise molecular mechanism linking Orai1/STIM1 to RhoA and DLBCL migration. The elucidation of this mechanism could be particularly relevant for development of drugs targeting DLBCL dissemination.

## Supplementary Materials

The following are available online at http://www.mdpi.com/2072-6694/10/11/402/s1, Figure S1: SDF-1 provokes an intracellular Ca^2+^ response in the SU-DHL-4 DLBCL cell line involving intracellular Ca^2+^ pool mobilization and Orai1/STIM1 extracellular Ca^2+^ influx; Figure S2: Validation of pharmacological and shRNA strategies used to study Ca^2+^ signaling in SUDHL4 and HLY1 cell lines; Figure S3: Involvement of CXCR4 in SDF-1-induced Ca^2+^ and migratory responses in the SU-DHL-4 and HLY-1 DLBCL cell lines; Figure S4: Comparison of STIM1 expression, proliferation and dissemination of HLY-1 cells in intrahepatic xenograft mouse model.

## References

[1] Møller M.B., Pedersen N.T., Christensen B.E. (2004). Diffuse large B-cell lymphoma: clinical implications of extranodal versus nodal presentation—A population-based study of 1575 cases. Br. J. Haematol..

[2] Boussios S., Zerdes I., Vassou A., Bareta E., Seraj E., Papoudou-Bai A., Pavlidis N., Batistatou A., Pentheroudakis G. (2018). Extranodal diffuse large B-cell lymphomas: A retrospective case series and review of the literature. Hematol. Rep..

[3] Pals S.T., de Gorter D.J.J., Spaargaren M. (2007). Lymphoma dissemination: the other face of lymphocyte homing. Blood.

[4] Broxmeyer H.E. (2008). Chemokines in hematopoiesis. Curr. Opin. Hematol..

[5] Nie Y., Waite J., Brewer F., Sunshine M.-J., Littman D.R., Zou Y.-R. (2004). The role of CXCR4 in maintaining peripheral B cell compartments and humoral immunity. J. Exp. Med..

[6] Cyster J.G. (2003). Homing of antibody secreting cells. Immunol. Rev..

[7] Tokoyoda K., Egawa T., Sugiyama T., Choi B.-I., Nagasawa T. (2004). Cellular Niches Controlling B Lymphocyte Behavior within Bone Marrow during Development. Immunity.

[8] Chen J., Xu-Monette Z.Y., Deng L., Shen Q., Manyam G.C., Martinez-Lopez A., Zhang L., Montes-Moreno S., Visco C., Tzankov A. (2015). Dysregulated CXCR4 expression promotes lymphoma cell survival and independently predicts disease progression in germinal center B-cell-like diffuse large B-cell lymphoma. Oncotarget.

[9] Teicher B.A., Fricker S.P. (2010). CXCL12 (SDF-1)/CXCR4 Pathway in Cancer. Clin. Cancer Res..

[10] Luo J., Li D., Wei D., Wang X., Wang L., Zeng X. (2017). RhoA and RhoC are involved in stromal cell-derived factor-1-induced cell migration by regulating F-actin redistribution and assembly. Mol. Cell. Biochem..

[11] Henschler R., Piiper A., Bistrian R., Möbest D. (2003). SDF-1alpha-induced intracellular calcium transient involves Rho GTPase signalling and is required for migration of hematopoietic progenitor cells. Biochem. Biophys. Res. Commun..

[12] Zuccolo E., Di Buduo C., Lodola F., Orecchioni S., Scarpellino G., Kheder D.A., Poletto V., Guerra G., Bertolini F., Balduini A. (2018). Stromal Cell-Derived Factor-1α Promotes Endothelial Colony-Forming Cell Migration Through the Ca2+-Dependent Activation of the Extracellular Signal-Regulated Kinase 1/2 and Phosphoinositide 3-Kinase/AKT Pathways. Stem Cells Dev..

[13] Iamshanova O., Fiorio Pla A., Prevarskaya N. (2017). Molecular mechanisms of tumour invasion: Regulation by calcium signals. J. Physiol..

[14] Feske S., Wulff H., Skolnik E.Y. (2015). Ion channels in innate and adaptive immunity. Annu. Rev. Immunol..

[15] Feske S., Gwack Y., Prakriya M., Srikanth S., Puppel S.-H., Tanasa B., Hogan P.G., Lewis R.S., Daly M., Rao A. (2006). A mutation in Orai1 causes immune deficiency by abrogating CRAC channel function. Nature.

[16] Yeromin A.V., Zhang S.L., Jiang W., Yu Y., Safrina O., Cahalan M.D. (2006). Molecular identification of the CRAC channel by altered ion selectivity in a mutant of Orai. Nature.

[17] Chen Y.-F., Chiu W.-T., Chen Y.-T., Lin P.-Y., Huang H.-J., Chou C.-Y., Chang H.-C., Tang M.-J., Shen M.-R. (2011). Calcium store sensor stromal-interaction molecule 1-dependent signaling plays an important role in cervical cancer growth, migration, and angiogenesis. Proc. Natl. Acad. Sci. USA.

[18] Chen Y.-T., Chen Y.-F., Chiu W.-T., Wang Y.-K., Chang H.-C., Shen M.-R. (2013). The ER Ca2+ sensor STIM1 regulates actomyosin contractility of migratory cells. J. Cell Sci..

[19] Jardin I., Rosado J.A. (2016). STIM and calcium channel complexes in cancer. BBA–Mol. Cell Res..

[20] Friedl P. (2004). Prespecification and plasticity: Shifting mechanisms of cell migration. Curr. Opin. Cell Biol..

[21] Takemura H., Hughes A.R., Thastrup O., Putney J.W. (1989). Activation of calcium entry by the tumor promoter thapsigargin in parotid acinar cells. Evidence that an intracellular calcium pool and not an inositol phosphate regulates calcium fluxes at the plasma membrane. J. Biol. Chem..

[22] Stolwijk J.A., Zhang X., Gueguinou M., Zhang W., Matrougui K., Renken C., Trebak M. (2016). Calcium Signaling is Dispensable for Receptor-Regulation of Endothelial Barrier Function. J. Biol. Chem..

[23] Kondratska K., Kondratskyi A., Yassine M., Lemonnier L., Lepage G., Morabito A., Skryma R., Prevarskaya N. (2014). Orai1 and STIM1 mediate SOCE and contribute to apoptotic resistance of pancreatic adenocarcinoma. Biochim. Biophys. Acta.

[24] Andrique L., Poglio S., Prochazkova-Carlotti M., Kadin M.E., Giese A., Idrissi Y., Beylot-Barry M., Merlio J.-P., Chevret E. (2016). Intrahepatic Xenograft of Cutaneous T-Cell Lymphoma Cell Lines: A Useful Model for Rapid Biological and Therapeutic Evaluation. Am. J. Pathol..

[25] Vacher P., Vacher A.-M., Pineau R., Latour S., Soubeyran I., Pangault C., Tarte K., Soubeyran P., Ducret T., Bresson-Bepoldin L. (2015). Localized Store-Operated Calcium Influx Represses CD95-Dependent Apoptotic Effects of Rituximab in Non-Hodgkin B Lymphomas. J. Immunol..

[26] Liu X.Q., Fourel L., Dalonneau F., Sadir R., Leal S., Lortat-Jacob H., Weidenhaupt M., Albiges-Rizo C., Picart C. (2017). Biomaterial-enabled delivery of SDF-1α at the ventral side of breast cancer cells reveals a crosstalk between cell receptors to promote the invasive phenotype. Biomaterials.

[27] Agle K.A., Vongsa R.A., Dwinell M.B. (2010). Calcium Mobilization Triggered by the Chemokine CXCL12 Regulates Migration in Wounded Intestinal Epithelial Monolayers. J. Biol. Chem..

[28] Prevarskaya N., Skryma R., Shuba Y. (2011). Calcium in tumour metastasis: new roles for known actors. Nat. Rev. Cancer.

[29] Wei S.H., Parker I., Miller M.J., Cahalan M.D. (2003). A stochastic view of lymphocyte motility and trafficking within the lymph node. Immunol. Rev..

[30] Cahalan M.D., Chandy K.G. (2009). The functional network of ion channels in T lymphocytes. Immunol. Rev..

[31] Palmesino E., Moepps B., Gierschik P., Thelen M. (2006). Differences in CXCR4-mediated signaling in B cells. Immunobiology.

[32] Brandes M., Legler D.F., Spoerri B., Schaerli P., Moser B. (2000). Activation-dependent modulation of B lymphocyte migration to chemokines. Int. Immunol..

[33] Borowiec A.-S., Bidaux G., Tacine R., Dubar P., Pigat N., Delcourt P., Mignen O., Capiod T. (2014). Are Orai1 and Orai3 channels more important than calcium influx for cell proliferation?. BBA–Mol. Cell Res..

[34] Vrenken K.S., Jalink K., van Leeuwen F.N., Middelbeek J. (2016). Beyond ion-conduction: Channel-dependent and -independent roles of TRP channels during development and tissue homeostasis. Biochim. Biophys. Acta.

[35] Genova T., Grolez G.P., Camillo C., Bernardini M., Bokhobza A., Richard E., Scianna M., Lemonnier L., Valdembri D., Munaron L. (2017). TRPM8 inhibits endothelial cell migration via a non-channel function by trapping the small GTPase Rap1. J. Cell Biol..

[36] Shinde A.V., Motiani R.K., Zhang X., Abdullaev I.F., Adam A.P., González-Cobos J.C., Zhang W., Matrougui K., Vincent P.A., Trebak M. (2013). STIM1 controls endothelial barrier function independently of Orai1 and Ca^2+^ entry. Sci. Signal..

[37] Mele S., Devereux S., Ridley A.J. (2014). Rho and Rap guanosine triphosphatase signaling in B cells and chronic lymphocytic leukemia. Leuk. Lymphoma.

[38] Heasman S.J., Carlin L.M., Cox S., Ng T., Ridley A.J. (2010). Coordinated RhoA signaling at the leading edge and uropod is required for T cell transendothelial migration. J. Cell Biol..

[39] Srikanth S., Gwack Y. (2012). Orai1, STIM1, and their associating partners. J. Physiol..

[40] Lopez J.J., Albarran L., Gómez L.J., Smani T., Salido G.M., Rosado J.A. (2016). Molecular modulators of store-operated calcium entry. Biochim. Biophys. Acta.

[41] Pan Y.-R., Chen C.-C., Chan Y.-T., Wang H.-J., Chien F.-T., Chen Y.-L., Liu J.-L., Yang M.-H. (2018). STAT3-coordinated migration facilitates the dissemination of diffuse large B-cell lymphomas. Nat. Commun..

[42] Middle S., Coupland S.E., Taktak A., Kidgell V., Slupsky J.R., Pettitt A.R., Till K.J. (2015). Immunohistochemical analysis indicates that the anatomical location of B-cell non-Hodgkin’s lymphoma [is determined by differentially expressed chemokine receptors, sphingosine-1-phosphate receptors and integrins. Exp. Hematol. Oncol..

[43] Cheng C.-L., Su Y.-C., Chao T.-Y., Lin C.-W., Chou S.-C., Yao M., Kuo S.-H., Yu S.-C. (2018). Intralymphatic Spread is a Rare Finding Associated with Poor Prognosis in Diffuse Large B-Cell Lymphoma with Extranodal Involvements. Am. J. Surg. Pathol..

[44] Rey C., Faustin B., Mahouche I., Ruggieri R., Brulard C., Ichas F., Soubeyran I., Lartigue L., De Giorgi F. (2016). The MAP3K ZAK, a novel modulator of ERK-dependent migration, is upregulated in colorectal cancer. Oncogene.

