# Supplementary material for: Calcium Independent Effect of Orai1 and STIM1 in Non-Hodgkin B Cell Lymphoma Dissemination
Source: Cancers (Basel). 2018 Oct 26;10(11):402. doi: 10.3390/cancers10110402 (PMC6267368; doi:10.3390/cancers10110402)
Supplement: Supplementary file 1 [file cancers-10-00402-s001.pdf]

Supplementary material

Calcium Independent Effect of Orai1 and STIM1 in Non-Hodgkin B Cell Lymphoma Dissemination

Simon Latour, Isabelle Mahouche, Floriane Cherrier, Lamia Azzi-Martin, Valérie Velasco, Pierre Soubeyran, Jean-Philippe Merlio, Sandrine Poglio and Laurence Bresson-Bepoldin

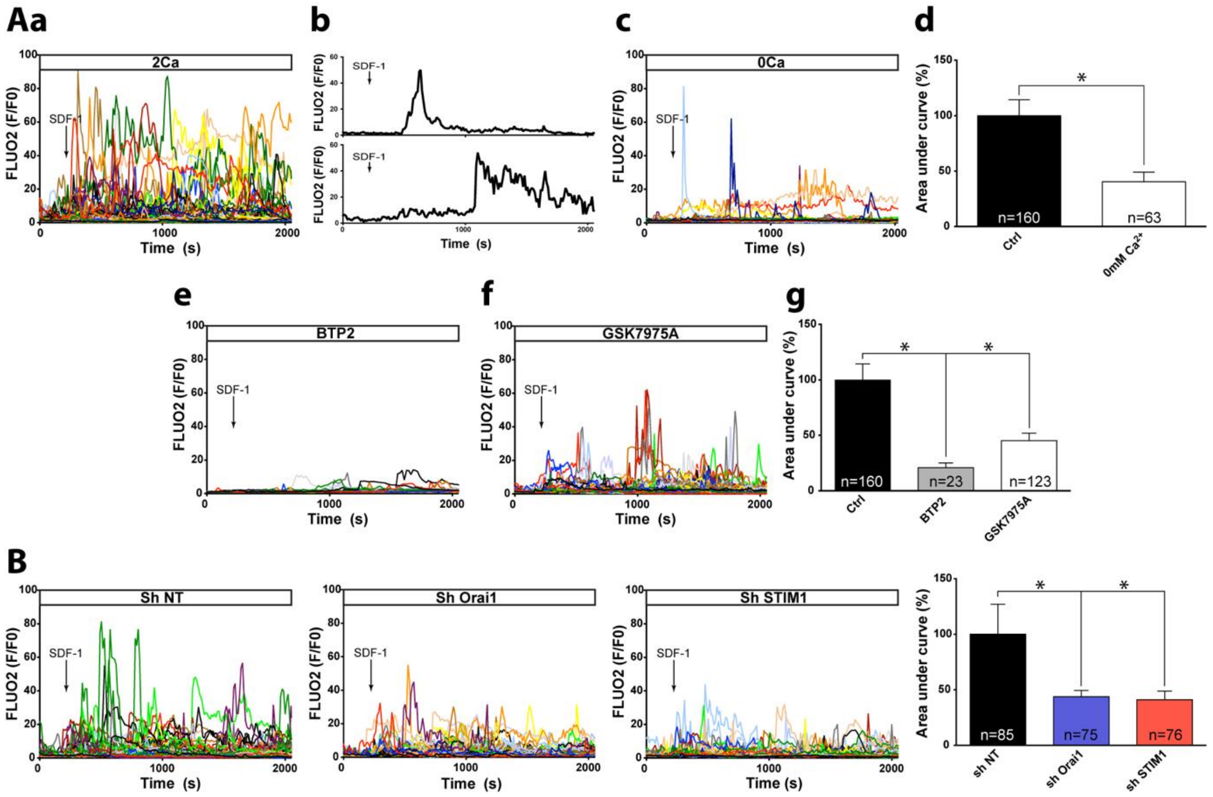

Figure S1. SDF-1 provokes an intracellular Ca^{2+} response in the SU-DHL-4 DLBCL cell line involving intracellular Ca^{2+} pool mobilization and Orai1/STIM1 extracellular Ca^{2+} influx. Ca^{2+} responses to SDF-1 (100 ng/mL) were measured using Fluo2-LR-AM Ca^{2+} dye and recorded by video microscopy (Zeiss LSM 510) using ×25 objective. Black arrows indicate SDF-1 addition. Each trace represents the response of one cell and data are representative of at least 3 independent experiments. Typical response of unique cell (peak or peak follow by sustained plateau phase) are present as example (**Ab**). Data were processed using GraphPad prism. (**A**) Pharmacological characterization of SDF-1-induced Ca^{2+} increase. Cells were recorded in extracellular saline solution (HBSS) containing 2 mM Ca^{2+} (2 Ca, (**Aa**)) or in Ca^{2+}-free HBSS (0 Ca, (**Ac**)). Cells were pre-incubated with BTP2 (**Ae**) or GSK7975A (**Af**) at 10 µM for 30 min and recorded in 2 mM Ca^{2+} HBSS containing inhibitors. (**Ad**,**Ag**) Histograms represent areas under curves (AUC) calculated, under various recording conditions, between the application time of SDF-1 and t = 2050 s, and normalized compared to control (2 Ca or shNT). Data are expressed as mean ± SEM, * $p < 0.05$. (**B**) Effect of Orai1 or STIM1 expression knock-down on SDF-1-induced Ca^{2+} response. The stable modified SU-DHL-4 cell line established after lentiviral transduction with plasmid containing non targeting shRNA (shNT), shRNA against Orai1 or STIM1 were recorded in extracellular saline solution (HBSS) containing 2 mM Ca^{2+}.

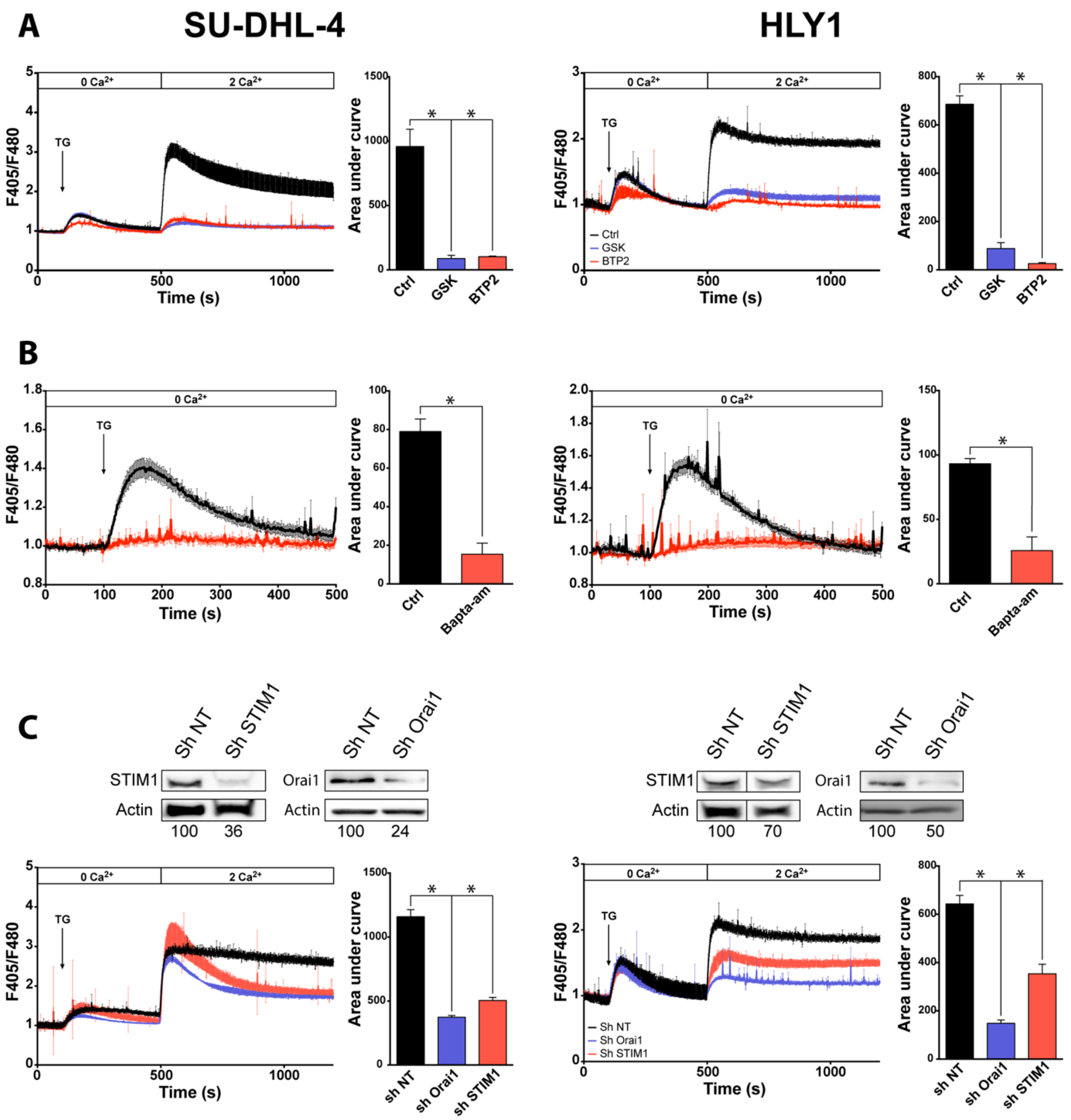

Figure S2. Validation of pharmacological and shRNA strategies used to study Ca^{2+} signaling in SU-DHL-4 (left) and HLY1 (right) cell lines. Ca^{2+} responses to TG (1 µM) were recorded on cell population using a Hitachi F-2500 spectrofluorimeter. Cells were loaded with Indo1-AM in Ca^{2+}-free HBSS, then at 500 s, 2mM Ca^{2+} were added in the extracellular medium to visualize SOCE. Data were processed using GraphPad prism. The data represent the result of 3 independent experiments. Histograms represent areas under curves (AUC) calculated between the application of TG to 500 s for measuring intracellular pool released by TG and between the application of 2 mM Ca^{2+} (at 500 s) and the end of recordings (at 1200 s) for measuring SOCE. Data are expressed as mean ± SEM, * $p < 0.05$. (**A**) Effect of GSK7975A (GSK, 10 µM) and BTP2 (10 µM) on SOCE induced by application of Thapsigargin (TG). (**B**) Effect of BAPTA-AM (5 µM) on intracellular Ca^{2+} release induced by TG. (**C**) Validation of SU-DHL-4 and HLY-1 cell lines infected with non-targeting or Orai1- or STIM1 targeting shRNA lentiviruses. Effect of shOrai1 and shSTIM1 on SOCE induced by TG. Upper panels: Orai1 and STIM1 expression level was evaluated by western blot. β-actin was used as a loading control.

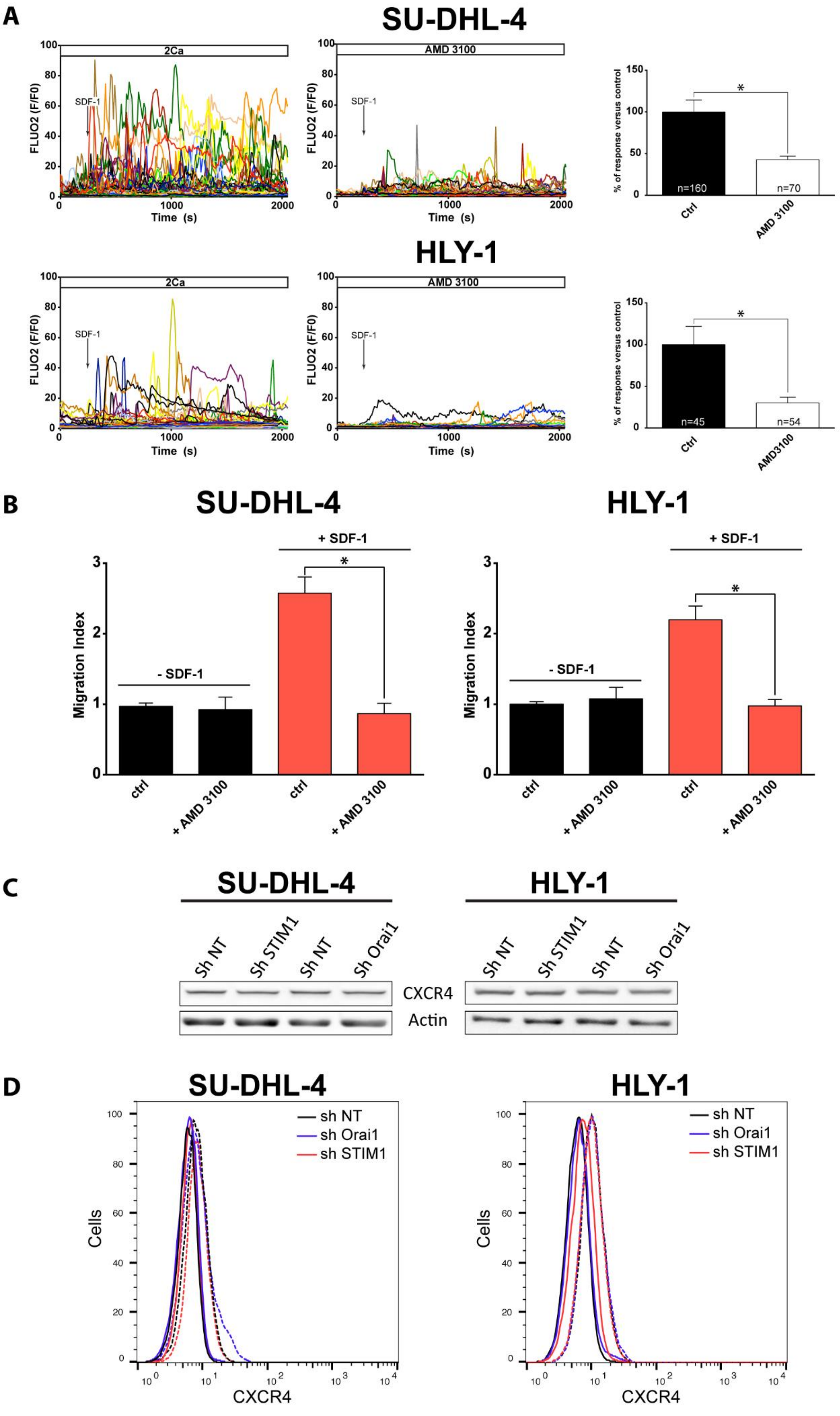

Figure S3. Involvement of CXCR4 in SDF-1-induced Ca^{2+} and migratory responses in the SU-DHL-4 and HLY-1 DLBCL cell lines. (**A**) SDF-1-induced Ca^{2+} response is dependent on CXCR4 activation.

Ca^{2+} responses to SDF-1 (100 ng/mL) were measured as described in Figure S1. Cells were pre-incubated or not (2Ca) with AMD3100 (5 μM) for 30 min and recorded in 2 mM Ca^{2+} HBSS with or without inhibitor. Histograms represent areas under curves (AUC) calculated, under various recording conditions, between the application time of SDF-1 and t = 2000 s, and normalized compared to control (2 Ca). Data are expressed as mean±SEM, * $p < 0.05$. (**B**) SDF-1-induced SU-DHL-4 and HLY-1 migration is dependent on CXCR4 activation. Cell migration was assessed as described in Figure 2. To test the effect of AMD3100 on chemotaxis induced by SDF-1, cells were pre-treated for 20 min with AMD3100 (150 nM) before loading onto upper transwell chambers maintained in medium during the experiment. Results are presented as migration index obtained by dividing the number of migrated cells in the studied condition by the number of migrated cells in the absence of chemoattractant (control). Histograms represent mean ± SEM from at least 3 independent experiments, * $p < 0.05$. (**C**) Total CXCR4 expression is not altered by knock-down of Orai1 or STIM1 in DLBCL cell lines. The stable modified SU-DHL-4 and HLY-1 cell lines established after lentiviral transduction with plasmid containing non-targeting shRNA (shNT), shRNA against Orai1 or STIM1 were harvested and lysed and the expression level of CXCR4 was evaluated by immunoblot analysis. β-actin was used as loading control. (**D**) Membrane CXCR4 expression is not altered by knock-down of Orai1 or STIM1 in DLBCL cell lines. SU-DHL-4 and HLY-1 cells expressing sh NT or sh Orai1 or sh STIM1 were stained with PE/Cy5 anti-human CXCR4 (dashed lines) or PE/Cy5 isotype control (lines). Fluorescence intensity was measured by flow cytometry.

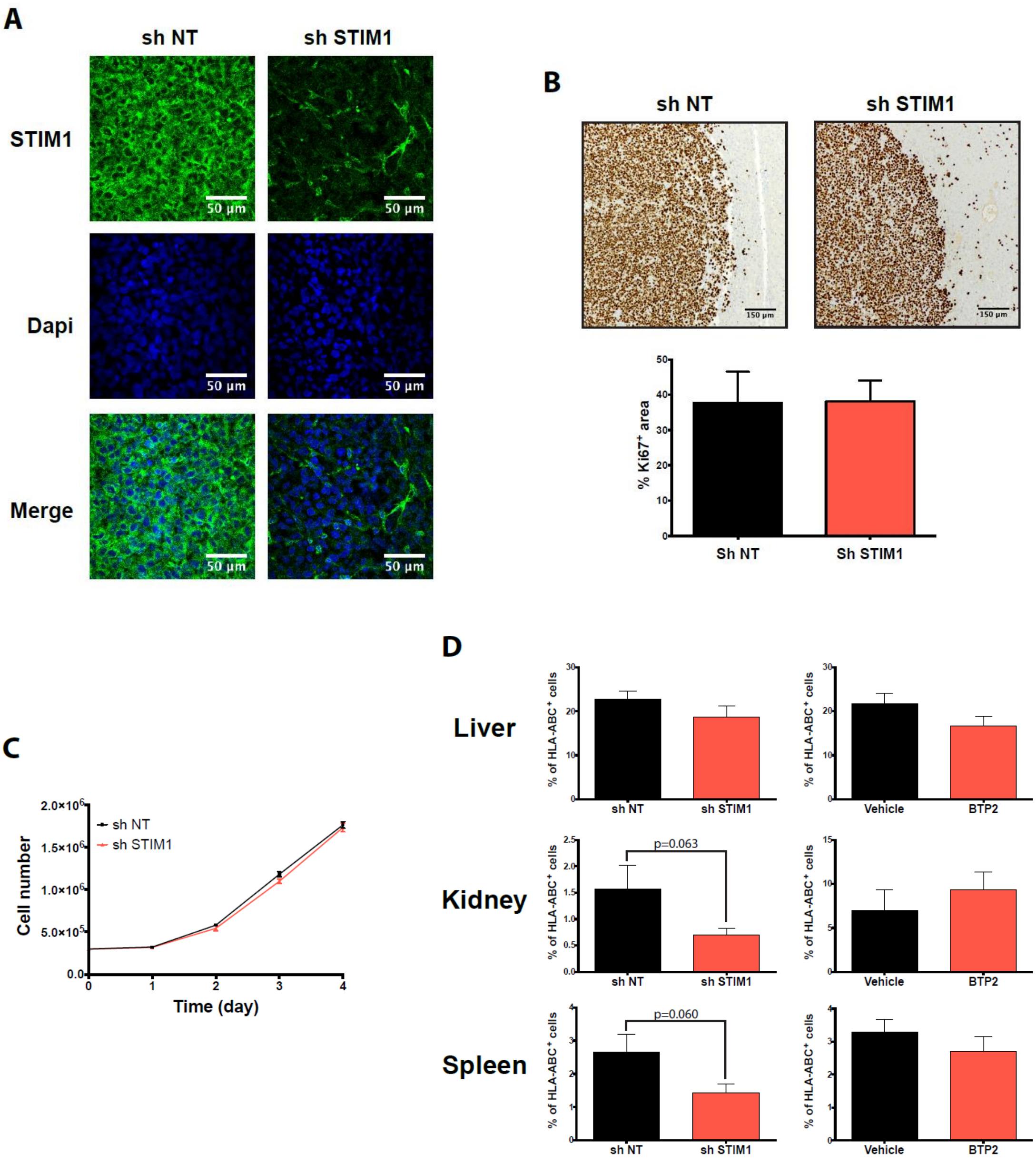

Figure S4. Comparison of STIM1 expression, proliferation and dissemination of HLY-1 cells in intrahepatic xenograft mouse model. (**A**) STIM1 under expression is maintained in mice xenografted with HLY-1 shSTIM1. Four weeks after intrahepatic xenograft of HLY-1 shNT or HLY1shSTIM1 cells, animals were sacrificed and formalin-fixed, paraffin-embedded liver sections were deparaffinized, and immunofluorescence was performed using rabbit polyclonal anti-human STIM1 antibody (HPA012123) revealed by Alexa 488-conjugated donkey anti-rabbit polyclonal antibody. Images were acquired using a Zeiss LSM 510 meta confocal microscope (Zeiss, Göttingen, Germany) with a 25× objective. Scale bar=50 µm. (**B**) No modification of cell proliferation in tumor induced by HLY-1 shNT or shSTIM1 cells. Liver sections obtained as described as were immunostained with monoclonal mouse anti-human Ki67 clone MIB-1. Images were captured with a Nikon Eclipse Ci microscope equipped with a Plan Fluor 10× 0.3 NA objective. Scale bars = 150 µm. Histograms represent the quantification of Ki-67 positive area in livers of mouse grafted with either HLY-1 shNT or shSTIM1 cells. (**C**) Under-expression of STIM1 do not modify cell proliferation *in vitro*. 3×10^5 HLY-1 cells expressing shNT or shSTIM1 were seed and count was done every day during 4 days. Datas represent mean ± SEM from 3 independent experiments. (**D**) DLBCL dissemination was quantified by flow cytometry in mice xenografted with shNT or sh STIM1, or in mice xenografted with HLY1 and treated with vehicle or BTP2 (12 µg/kg), three times per week. After mice sacrifice, the organs were removed, dissociated, immunostained with HLA-ABC-PECy7 antibody and analyzed by flow cytometry.

Histograms represent the quantification of HLA-ABC positive cells. Data are represented as mean ± SEM (n = 10).

© 2018 by the authors. Submitted for possible open access publication under the terms and conditions of the Creative Commons Attribution (CC BY) license (http://creativecommons.org/licenses/by/4.0/).
